# The Concept of Chronic Edema—A Neglected Public Health Issue and an International Response: The LIMPRINT Study

**DOI:** 10.1089/lrb.2018.0085

**Published:** 2019-04-22

**Authors:** Christine Moffatt, Vaughan Keeley, Isabelle Quéré

**Affiliations:** ^1^ School of Social Sciences, Nottingham Trent University, Nottingham, United Kingdom.; ^2^ Montpellier Medecine Vasculaire, EA2992, Universite Montpellier I, CHU Saint Eloi, Montpellier, France.; ^3^ Copenhagen Wound Healing and Lymphoedema Centre, Bisperberg University Hospital, Copenhagen, Denmark.; ^4^ Lymphoedema Service, Royal Derby Hospital, Derby, United Kingdom.

**Keywords:** lymphedema, lymphoedema, chronic edema, prevalence, incidence, LIMPRINT

## Abstract

Lymphedema has always been a neglected global health care problem. A central requirement for the development of any chronic disease is the clear use of public health definitions that can be used internationally to define populations. The term “lymphedema” has historically been defined as either primary, resulting from failure of lymphatic development, or secondary, following damage to the lymphatics (e.g., cancer treatment, injury, or filariasis). Attempts to integrate causes of edema arising from damage to the venous system or the effects of gravity, immobility, and systemic disease have rarely been integrated. More recently, the prominent role of the lymphatics in tissue fluid homeostasis in all forms of chronic edema has been recognized. These advances led to the development of the term: “Chronic edema: a broad term used to describe edema, which has been present for more than three months.” It can be considered an umbrella term that includes not only conventional “lymphedema” but also chronic swelling, which may have a more complex cause. This definition has been adapted in the international epidemiology study (LIMPRINT) that identified people throughout the health and social care systems in participating countries. Clearer definitions will allow for examination of this important public health problem that is likely to escalate given the projections of an aging population with multiple comorbidities. It will be possible to define both the hidden mortality and morbidity associated with complications, such as cellulitis and the impact on health-related quality of life. This evidence is urgently required to lobby for increased resource and effective health care in an increasingly competitive health care arena in which more established conditions have greater priority and funding.

## The International Lymphedema Framework

The mission of the International Lymphedema Framework (ILF) is to improve the care of people with lymphedema and related disorders worldwide, which can only be carried out when the problem is recognized as a neglected public health problem that is largely ignored. The ILF, a charitable organization with an international vision, has recognized since its inception that there is a lack of global awareness of the size and impact of people suffering with different forms of lymphedema and related disorders.^1^ This is an extraordinary and unacceptable situation given the clinical and personal significance for those affected by the condition and the decades of research that have occurred in this field. Without this fundamental information, resources are unlikely to ever be allocated for management, and further, the global challenges of lack of reimbursement and low investment will maintain the status quo.

## The ILF Response: LIMPRINT

The concept of designing and implementing an international epidemiology study to address these issues emerged as a key strategic aim for the ILF. There were many methodological challenges faced in creating systems that could be used in countries with a diverse range of health care settings. Such a vision was not for the weak hearted; it required a huge commitment from many different stakeholders. At the very heart of this initiative was the belief in the power of partnership working; the ethos of the ILF and part of its motto—belong together.

The development of LIMPRINT as an international epidemiology study required the engagement of multiple stakeholders including: all national lymphedema frameworks; clinicians; academics; patient organizations; the medical device industry; and other organizations, for example, charities. It has required a commitment from a diverse range of countries and individual sites who have participated to provide resources and to drive projects. All these initiatives have received little or no direct funding but have sought local research opportunities and benefited from effective academic leadership. LIMPRINT has formed the largest international epidemiology project on lymphedema to date.

## Who Is Affected by Lymphedema? The Myth of a Rare Disorder

Lymphedema has always been a neglected area of health care. This has largely been because it has been misunderstood and thought to be a rare condition. In many parts of the world such as India and Africa, it is recognized as a neglected tropical disorder caused by filariasis, a parasitic condition spread by mosquitoes.^2^ In western populations, the causes are very different^3–6^ Nevertheless, there is a huge hidden burden of morbidity that spans all afflicted populations irrespective of where they are found across the world. Additionally, there is a failure to recognize the hidden mortality associated with complications such as cellulitis and a nihilistic view that no effective treatment exists. These beliefs have led to a lack of investment in service provision and research into the causes, treatment, and management. All these assumptions are fundamentally incorrect and are what the ILF and the other organizations dedicated to improving care are trying to challenge.

## Children with Lymphedema: A Truly Rare Condition

Lymphedema occurring in children and adolescents is correctly defined as a rare condition.^7^ Despite this, we have little international epidemiology on how many children are afflicted as most countries do not collect this information. This compounds the suffering for children and their families who require accurate diagnosis, genetic screening, and appropriate treatment within their own countries. For some, they are forced to seek help from international experts and travel abroad for treatment. The ILF recognizes the importance of this group and is seeking to develop an international response to address this. The determination of the genetic causes of lymphedema is a rapidly developing field of research with the identification of new causes for a proportion of those affected. This offers the hope of targeted interventions and a possible cure. Research is of critical importance since some secondary lymphedema may have an underlying genetic predisposition, impacting on the risk of its development following chronic infection, tissue damage, or surgical or other interventions.

## Adults with Lymphedema: A Heterogeneous Population

Adults with chronic edema are found in many parts of the health and social care systems.^8,9^ Despite this, professional knowledge is often poor leading to a lack of diagnosis and treatment. Both patients and professionals frequently ignore the early presentation of swelling until complications such as irreversible tissue changes, chronic wounds, cellulitis, or loss of function occur.

In many developed countries, the focus of lymphedema management and research has been on that resulting from the treatment of cancer. It is only in recent years that other causes have been given greater attention, and it is now recognized that cancer-related lymphedema constitutes only a relative minority of the total cases.

## Lymphedema or Chronic Edema: The Importance of Definition

A central requirement for the development of any chronic disease is the clear use of public health definitions that can be used internationally to define the different patient populations. Only then can the risk factor profiles be identified, and targeted treatments developed. This has been a major problem for the field of lymphedema and its related disorders. It has led to confusion for the general public and professionals and has potentially affected its recognition as a health care problem.

Confusion over who can “claim” to be suffering from lymphedema is such a significant issue that it underpins the reimbursement and provision of care in many countries. In some, payment is only provided for those with lymphedema linked to cancer or those with a primary form confirmed by clinical investigation. In countries where lymphedema is a neglected tropical disease, there is a tendency for all those living in these areas to be classified as suffering from filariasis-related lymphedema, even though many will have other forms of lymphedema.

### The changing perspectives on definition

The term “lymphedema” has historically been defined as edema, which develops as a result of failure of lymphatic drainage either through problems in lymphatic development (primary lymphedema) or through damage to the lymphatics (secondary lymphedema; e.g., following cancer treatment). Edema arising from venous disease is not always considered to be a secondary type of lymphedema, although there is evidence of lymphatic failure in chronic venous disease with edema where the term “phlebolymphedema” is often used.^5^

### Complex patient profiles

In many clinical situations such as in the elderly with multiple comorbidities, many factors may contribute to the etiology of chronic swelling, for example, immobility, heart failure, chronic venous hypertension, and drugs. A few health care professionals would consider this swelling to be lymphedema. However, this type of chronic swelling does involve failure of lymphatic drainage with significant resulting morbidity.

Furthermore, in some types of lymphedema, which have previously been considered to be purely due to problems with lymphatic drainage, for example, breast cancer-related lymphedema, and some types of primary lymphedema, for example, lymphedema–distichiasis (in which there are malfunctioning lymphatic and venous valves), there is growing evidence of a more complex cause including a venous component and genetic predisposition to its development.^10,11^

The recognition of the complex patients presenting to a national United Kingdom specialist service led to the development of the term “chronic edema.” This was used in the prevalence study carried out in 2003.^8^ The definition used in this study is defined below:

“Chronic oedema is a broad term used to describe oedema which has been present for more than three months and involves one or more of the following areas: limbs, hands/feet, upper body (breast/chest wall, shoulder, back), lower body (buttocks, abdomen), genital (scrotum, penis, vulva), head, neck or face.”

Thus, “chronic edema” can be considered to be an umbrella term that includes not only conventional “lymphedema” but also chronic swelling, which may have a more complex cause.

Chronic edema therefore includes:

- Lymphedema (primary and secondary)
- Venous edema
- Chronic swelling due to immobility
- Edema related to advanced cancer
- Chronic swelling associated with lipedema
- Chronic swelling related to obesity
- Chronic swelling associated with rare vascular malformations such as Klippel–Trenaunay syndrome.

### New understanding of the physiology

In recent years, our understanding of the physiology of tissue fluid formation and drainage has developed further and supports the concept that the lymphatics are involved in all forms of chronic edema.^12^

The Starling model proposed that fluid flow out of capillaries into the tissues was governed by net outcome of opposing pressures across the capillary wall. The pressures concerned are the hydrostatic pressure and the colloid osmotic (oncotic) pressure gradients. The flow rate is also governed by the permeability of the capillary wall. The hydrostatic pressure gradient is the physical pressure inside the capillary compared with that outside. The colloid osmotic pressure arises from the attraction of water by proteins and therefore the pressure gradient is due to the difference in protein concentration between the plasma and the tissue fluid.

In the original model, measurements of these pressures available at that time suggested that there was an outflow of fluid from the arteriolar end of the capillary and reabsorption of fluid into the venous end of the capillary, with only ∼10% of the tissue fluid being drained through the lymphatic system.

A more recent, more sophisticated understanding of the ultrastructure of the capillaries and more accurate measurements of the various pressures involved has led to a revision of these ideas. It is now thought that in the steady state, in most capillary beds, there is net outflow of fluid all along the capillary with no reabsorption at the venous end. This means that all the excess capillary filtrate and macromolecules in the interstitial space are taken up by the lymphatics. This gives the lymphatics a much greater role in tissue fluid homeostasis than previously understood.^12^

Edema arises when capillary filtration exceeds lymphatic drainage. If lymphatic drainage is reduced and capillary filtration is normal, edema develops (known as lymphedema if persistent/chronic). If capillary filtration is increased, then the lymphatics drain more fluid to prevent edema formation. In this situation, edema only develops when capillary filtration exceeds the maximum capacity of the lymphatics to drain fluid. Thus, it can be argued that all edema has a lymphatic component, whether it is due primarily to a lymphatic problem or to other factors, which cause an increase in capillary filtration.

It could therefore be argued that all chronic edema should be considered to be lymphedema. In this case, all the more complex types of chronic edema (e.g., that due to venous disease and advanced cancer) would be considered to be types of secondary lymphedema.

From a clinical viewpoint, whichever term is used, chronic edema or a broader understanding of the term “lymphedema,” it is still important to consider the underlying factors, which may be causing the swelling as this may influence treatment. Therefore, the term “chronic edema” is a useful umbrella term and should be used in public health studies but in itself it does not define the underlying cause(s) and is not a diagnosis.

When considering studies of prevalence, it is again important that the broader meaning of lymphedema or the term “chronic edema” is used synonymously to encompass the more complex causes of chronic swelling. In publications of prevalence studies to date, some use the term “chronic edema” and others the term “chronic edema/lymphedema.”^8,13–15^

### Methodology for determining prevalence

How common a chronic condition is can be determined by measuring the prevalence of the condition in the population at one point in time (point prevalence). Crude prevalence is a measure of all people with the condition at a single time point. This does not take into account any variation that may occur in the population such as age or gender. Measuring differences in prevalence in different age groups allows standardization of the prevalence calculation. This in turn enables comparisons to be made between the prevalence in different populations taking account of the influence of different age distributions. Comparison between prevalence data from different settings is also facilitated by defining the population in which the prevalence was measured and having a consistent definition of the condition concerned.

To measure how common lymphedema is in a given country, the number of people with lymphedema in the population of that country would need to be determined at a given time point. Population-based studies are difficult to carry out from a practical point of view and are also expensive. Alternative methods can, however, yield useful information. One such method is to measure the number of people with lymphedema known to health care professionals in a given population. This assumes that all people with lymphedema are known to local health services, which, as already discussed, is unlikely given the lack of professional knowledge and public awareness.

Furthermore, in an ideal situation, there would be some form of routine data collection or register for those with lymphedema, which could be interrogated to determine the prevalence in each country using agreed definitions and standardized coding. Unfortunately, while in some countries this occurs, (particularly in those with insurance-based health care systems), this is not standardized internationally.

Therefore, case ascertainment methodology, which includes carrying out a survey of health care professionals in a given population, asking them for details of people known to them with chronic edema, in addition to prospective clinical assessment of patients within the health care systems, is an appropriate compromise that yields important results. It is this which forms the core of the LIMPRINT methodology.

This approach can also be used to determine the prevalence in smaller populations such as those with another chronic clinical condition where chronic swelling is known to occur, for example, multiple sclerosis.^4^ This still requires a database of those with the other chronic conditions, which is kept up-to-date to define the population (denominator) accurately.

Cross-sectional-based prevalence studies can be undertaken in defined health care settings such as hospitals or care homes where the population is fixed over a short time period. In these settings, a visiting team of researchers can determine the number of people and clinically assess them, thereby deriving an accurate estimation of the prevalence in that setting at that particular time point.

### The importance of incidence data

In some situations, the measurement of the incidence of lymphedema/the risk of developing lymphedema, for example, after breast cancer treatment, can also be valuable. Incidence measures the number of new cases over a given time period. In the case of breast cancer-related lymphedema, the condition most commonly develops within 2 years of the initial cancer treatment, but it is known that some women do not develop lymphedema until many years later even in the absence of recurrent disease. This means that in studies of incidence where the lifelong risk of developing breast cancer-related lymphedema is being determined, the time period of follow-up must be sufficiently long, for example more than 3 years to give the most accurate estimate.

Whether measuring prevalence or incidence, it is important that the diagnosis of “lymphedema” is defined and used consistently. This has been clearly illustrated in the case of lymphedema following breast cancer treatment where a number of different definitions of lymphedema judged by limb volume changes following surgery compared with preoperative measurements have been used. Using definitions of volume changes of 200 mL, 5% or 10% give very different estimates of incidence.^3^

### Epidemiological studies of chronic edema in the United Kingdom

The first study to use the definition of chronic edema was reported in 2003.^8^ The aims of the study were to determine the magnitude of the problem of chronic edema in health services within an urban area of London, United Kingdom, and to assess the likely impact of edema on use of health resources, employment, and patient's quality of life. The study used a questionnaire-based survey given to health professionals followed by an interview and clinical assessment in a random sample. Health professionals from dedicated lymphedema services, specific outpatient clinics, hospital wards, and community services (general practitioner clinics and district nurses) were contacted to provide information on patients from within the geographical area who were known to suffer with chronic edema of greater than 3 months duration.

Within the catchment area with a population of 619,000 people, 823 patients had chronic edema (crude prevalence 1.33/1000). Prevalence increased with age (5.4/1000 in those aged >65 years) and was higher in women (2.15 vs. 0.47/1000). Only 529 (64%) were receiving treatment, despite 2 specialist lymphedema clinics within the catchment area. Of 228 patients interviewed, 78% had edema lasting >1 year. Over the previous year, 64 of 218 (29%) had had an acute infection in the affected area, with 17 of 64 (27%) being admitted for intravenous antibiotics. The mean length of stay for this condition was 12 days, with an estimated mean cost of £2300 (2003 data). Edema caused time off work in >80% and affected employment status in 9%. Quality of life was below normal, with 50% experiencing pain or discomfort from their edema.

Using an extrapolation of these figures, it was estimated that at least 100,000 patients were suffering in the United Kingdom alone. However, it is acknowledged that this will be an underestimate of the true prevalence within the general population for the reasons described above.

This methodology was repeated over 10 years later in an urban population of the East Midlands in the United Kingdom.^9^ This cross-sectional study was carried out in Derby City (United Kingdom), which has a population of ∼247,100. Data were obtained from 10 sources, namely the inpatients of 1 acute and 1 community hospital, 1 specialist, and 3 nonspecialist outpatient clinics (dermatology, plastic surgery, and diabetic foot clinic), all community nursing services, general practices (*n* = 41), and nursing/residential homes (*n* = 26) in the catchment area.

Within the study population of Derby City residents, 971 patients were identified with chronic edema (estimated crude prevalence 3.93/1000, 95% [confidence interval 3.69–4.19]). The prevalence was highest among those aged 85 years or older (28.75/1000) and was higher among women (5.37/1000) than men (2.48/1000). The prevalence among hospital inpatients was 28.5%. Only five (3%) patients in the community population had edema related to cancer or cancer treatment. Patients with cancer-related lymphedema were usually treated by hospital-based services in Derby. Of the 304 patients identified with edema from the Derby hospitals or community health services, 121 (40%) had a concurrent leg ulcer.

### Study comparison

Data obtained from this study differ greatly from those obtained previously, even though the same methods were adopted. In the 2003 London study, the crude prevalence was approximately one third of that reported in 2016. When standardized to the population of England, this difference was reduced slightly to three times that observed in London, with adjusted rates for Derby City and South West London (4.15/1000 and 1.55/1000, respectively). It is unlikely that this difference can be attributed to methodological discrepancies or variations in the populations studied, as both samples were derived from an urban community. It is possible that differences in characteristics of the population other than age and gender such as obesity may be partially responsible for the higher prevalence. Other findings were comparable to the earlier London study, for example, the prevalence was much higher among women than men. It was also more prevalent among the obese and was highest among people older than 85 years.

Analysis of the data by site of swelling (*n* = 889) indicates that the proportion of patients with lower limb edema was much higher in Derby City than in the London study. This may have occurred because of a greater awareness of chronic edema in Derby, which may have led to a larger number of referrals of patients with lower limb edema to the Derby service compared with South West London. If this is the case, some of the difference in overall estimated prevalence could be attributed to greater identification of lower limb edema rather than a truly higher overall prevalence. This is unlikely, however, as both specialist services in Derby and South West London are well-known centers of excellence that have been established for many years.

### Hospital inpatient services

Nearly one third of the hospital inpatient population had chronic edema. This may be because a number of conditions are associated with its occurrence, and it can be caused by a variety of underlying pathophysiological mechanisms. This finding also dispels the commonly held belief that chronic edema is confined to those seen by community-based health services. While it is well recognized that many “community patients” have venous leg ulceration, this study highlights that many of these also have concurrent chronic edema, an association that has received scant attention previously.

### Obesity and chronic edema

The East Midlands data support the hypothesis that obesity is a common feature of patients with chronic edema seen by specialist services in western populations. A number of mechanisms have been postulated to explain this relationship. These include impaired lymphatic flow, chronic inflammation, elevated production of interstitial fluid, and reduced mobility in obesity. Obesity is also implicated in the development of chronic edema among people with cancer and those with other long-term conditions, particularly those who are wheelchair users.^16,17^

### Study limitations

One limitation of this study is that comprehensive data could not be obtained from General Practices as diagnostic codes have not been created in the United Kingdom health service. Poor recognition and limited knowledge may have limited the number of patients identified, particularly in nursing/residential home settings where the proportion of qualified staff is low. Of greater importance is the lack of awareness among the general population, as this limits the number of people who present to health services. It is very difficult to estimate the true percentage of the population as symptoms can develop at a relatively late stage. A major strength of this study is that patients were surveyed in all public health service settings available to Derby City residents and all nursing/residential homes.

## Conclusions

A systematic review has indicated that there is a dearth of population-based epidemiological studies to define the prevalence of chronic edema and little robust evidence of the cost and clinical effectiveness of different models of care.^18^

The studies undertaken in the United Kingdom created the impetus behind the concept of LIMPRINT with the possibility to assess the number of people with chronic edema in different health care services using an internationally agreed protocol of assessment and validated data collection methods. This will be discussed further in the following articles.
